# Effect of Salt Stress on Cytosine Methylation within GL2, An *Arabidopsis thaliana* Gene Involved in Root Epidermal Cell Differentiation. Absence of Inheritance in the Unstressed Progeny

**DOI:** 10.3390/ijms20184446

**Published:** 2019-09-10

**Authors:** Cecilia C. Beyrne, Norberto D. Iusem, Rodrigo M. González

**Affiliations:** 1 Instituto de Fisiología, Biología Molecular y Neurociencias (IFIByNE), CONICET, Buenos Aires C1428EGA, Argentina (C.C.B.) (N.D.I.); 2 Departamento de Fisiología, Biología Molecular y Celular (FBMC), Facultad de Ciencias Exactas y Naturales, Universidad de Buenos Aires, Buenos Aires C1428EGA, Argentina

**Keywords:** *Arabidopsis*, saline stress, DNA methylation, root epidermis, trichoblasts, Glabra-2

## Abstract

Methylation/demethylation of cytosines is an epigenetic strategy for transcriptional regulation, allowing organisms to rapidly respond and adapt to different stimuli. In this context, and using *Arabidopsis thaliana* as a plant model, we explored whether an environmental stress is sufficient to trigger a change in the methylation status of Glabra-2, a master gene associated with root epidermal cell differentiation. As this gene acts mainly in the epidermis in the root, we examined the stress-driven methylation levels specifically in that tissue. We focused on the stress caused by different salt concentrations in the growth medium. When testing the effect of 20 and 75 mM NaCl, we found that there is a significant decrease in the CG methylation level of the analyzed genomic region within the epidermis. Whereas this reduction was 23% in mildly stressed plants, it turned out to be more robust (33%) in severely stressed ones. Notably, this latter epigenetic change was accompanied by an increase in the number of trichoblasts, the epidermal cell type responsible for root hair development. Analysis of an eventual inheritance of epigenetic marks showed that the non-stressed progeny (F1) of stressed plants did not inherit—in a Lamarckian fashion—the methylation changes that had been acquired by the parental individuals.

## 1. Introduction

The two cell types that constitute the root epidermal cell layer are named trichoblasts and atrichoblasts. The former produce root hairs, a thin structure originated by apical cell growth, and have an active role in the uptake of water and nutrients from the soil. The abundance ratio between trichoblast and atrichoblasts is 1:2. Trichoblasts are always located over the junction of two cortical cells [1,2].

A high number of proteins play a role in root epidermis differentiation, composing a complex transcriptional network [3]. The signal pathway that results in epidermal cell differentiation is proposed to start with JKD (JACKDAW), a protein secreted by cortical cells. It is postulated that this protein interacts directly or indirectly with SCM (SCRAMBLED), a membrane receptor protein located in both types of epidermal cells. WER (WEREWOLF), a protein also present in both, in the undifferentiated atrichoblasts forms a complex with GL3 (GLABRA-3) and TTG1 (TRANSPARENT TESTA GLABRA-1) and this way promotes GL2 (GLABRA-2) gene expression. On the other hand, GL2 inhibits the expression of other genes directly involved in root hair development and the resulting cell is a differentiated atrichoblast. Conversely, in the future trichoblastic cells, WER is inhibited by SCM. GL3 and TTG1 form a complex with CPC (CAPRICE) and TRY (TRIPTYCHON), which inhibit the expression of GL2, resulting in a differentiated trichoblast [1].

*Arabidopsis* GL2 is a crucial gene for determination of the epidermal cell fate. Located in chromosome 1, it is 4484 bp in length with nine exons, eight introns and presents five splicing variants. GL2 is a homeobox leucin zipper protein, presenting a START lipid-binding domain and a nuclear localization signal. It has 747 amino acids and an isoelectric point of 6.48 [4]. Mutations in the GL2 gene leads to ectopic development of root hairs in positions where atrichoblast are normally present [5].

Epigenetics DNA modifications refer to changes that do not involve changes in its sequence [6]. In this context, acquisition of epigenetic marks allows for rapid favorable adaptive responses, independently of natural selection, which might take many generations [7]. One of these marks is the methylation of cytosines. In plants, cytosine methylation occurs in CG, CHG or CHH contexts (H denoting A, T or G nucleotides) [8]. Typically, methylation in the CG context is performed by MET1 (DNA METHYLTRANSFERASE-1) DNA methyltransferase and, in general, it represses gene transcription when located in the promoter region while having an opposite activating effect when present in the gene body [9]. On the other hand, methylation in CHG (performed by CMT3 (CHROMOMETHYLASE-3) methyltransferase) and CHH (by DRM2 (DOMAINS REARRANGED METHYLTRANSFERASE-2) methyltransferase and small RNAs) usually occurs in transposable elements, which otherwise would move along the genome [10,11]. Nevertheless, CHG and CHH can be found in the body of transcriptionally active genes [12,13].

As plants are sessile, they have to cope in situ with changing environment conditions thus being particularly affected by stresses like drought and salinity. In particular, saline stress leads to lower germination rates, shortening of the main root, low rosette size and leaf chlorosis. For example, sodium chloride concentrations of 50 mM or higher represent a severe stress condition as the phenotypes mentioned above become significant [14]. Mild or high salt concentrations in the medium could cause water stress in plants by hindering the uptake and retention of water, imitating this way the effect of a drought but also causing a toxicity effect given by the presence of sodium ions [15]. Obviously, these kind of abiotic stresses are harmful for human agronomical purposes as salt reduces water availability hence affecting crop yields. Saline soils comprise as much as 50–75 million acres around the world, increasing year after year [16,17].

GL2 changes its methylation levels as a consequence of genetic alterations as reported for three different *Arabidopsis* mutant lines [18]. Those results prompted us to explore whether this gene involved in epidermal cell fate—thus determining root hair development—gains or loses epigenetic marks when plants are exposed to environmental challenges. Furthermore, we searched for an eventual Lamarckian inheritance [19] of such epigenetic marks in the non-stressed progeny (F1).

## 2. Results

### 2.1. Analysis of GL2 Methylation in Salt-Stressed Plants 

**Figure 1:**
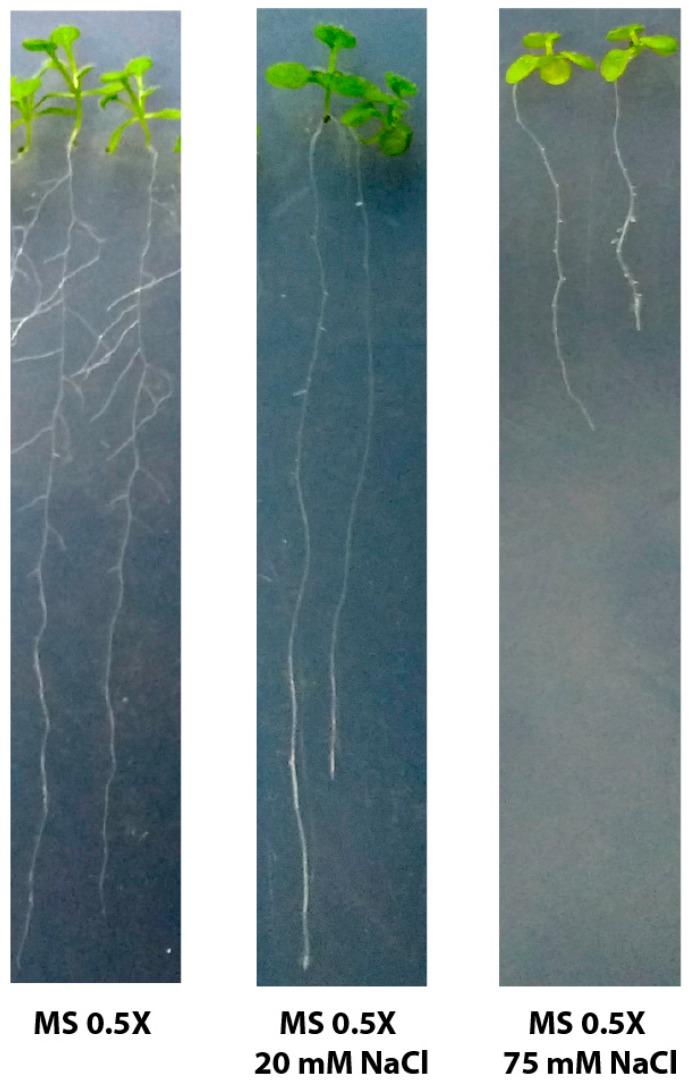
Photographs of three-week-old seedlings grown on MS (Murashige and Skoog) medium with no supplementation and supplemented with either 20 mM or 75 mM NaCl.

A portion of the GL2 gene, 339-bp long, including part of exon 6 and part of intron 6, was analyzed in stressed and non-stressed plants. This is the region amplified by either of the two primer pairs (long and nested amplicon) by means of PCR (Polymerase Chain Reaction), thus allowing us to compare between epidermis and whole root [18]. The standard sodium bisulphite protocol was employed using either epidermal-specific or non-epidermal-specific primers. It was interesting for us to study the effect of two different intensities of stress, namely a mild stress condition and a severe one. For this purpose, two assays were conducted by subjecting WT seeds to germination and growth in a medium with either a moderate concentration of NaCl (20 mM) or a higher one (75 mM). The latter can be convincingly considered a condition of severe stress judging from the altered phenotype observed in the individuals grown there (Figure 1). In the case of the plants mildly stressed, the main visible stress symptom was the inhibition of lateral roots, while in the severely stressed ones the overall plant health seen drastically affected, showing its main root extremely shorter, lack of development of secondary roots, smaller rosette area and a paler pigmentation of the leaves.

**Figure 2:**
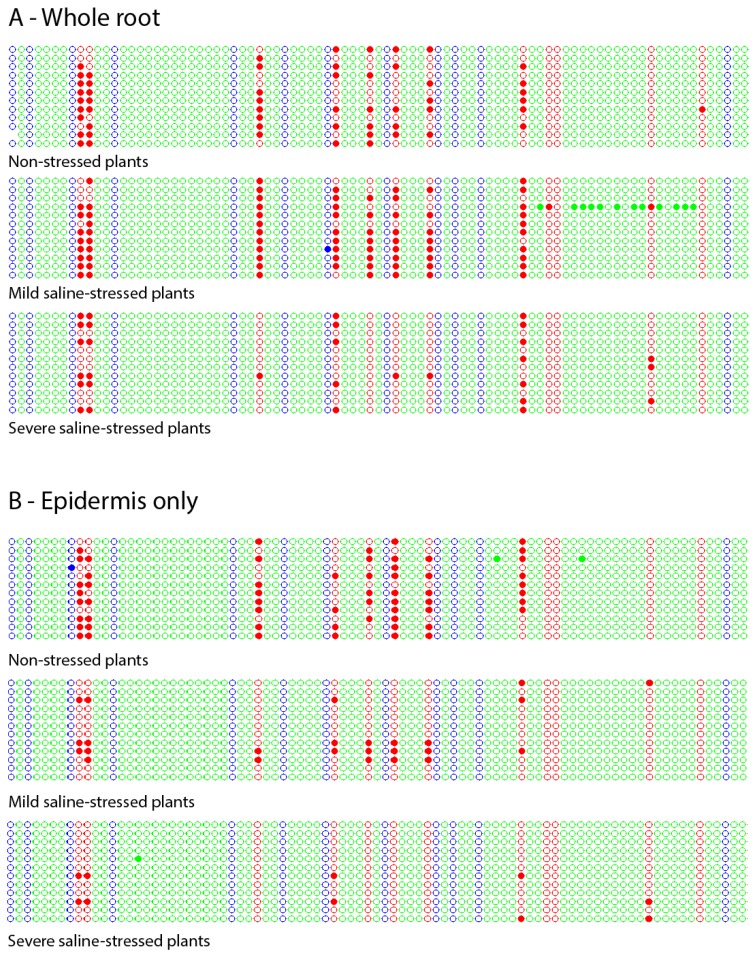
Kismeth dot plots [20] showing the methylation pattern of a region of GL2 in the root of non-stressed, mild-stressed and severe-stressed plants. (**A**) Total root. (**B**) Epidermis. Each line represents one clone, and each circle, one cytosine according to the order 5′–3′ (left–right) in which it appears in the DNA sequence. The colors blue, red and green correspond to cytosines located in the contexts -CHG-, -CG- and – CHH-, respectively. The filled circles indicate methylated cytosines, while the empty circles represent the unmethylated ones.

We analyzed the methylation pattern of GL2 in the root of plants grown under each of the three conditions, discriminating between epidermis and total root (Figure 2).

In all the cases, methylation was found, and mostly in the CG context. Interestingly, of the 12 cytosines in the CG context present in the analyzed sequence, there were some that never appeared methylated in any of the assays performed, both for the stressed and the non-stressed plants. Those cytosines were, in the order 5′-3′, the tenth (no matter which set of post-bisulphite PCR primers was used, which means total root), the ninth and the twelfth (these two only in epidermis). In contrast, there were others cytosines that appeared methylated in non-stressed plants but did not in severely stressed ones, namely the fifth (in total root), the third, the sixth and the seventh (only in epidermis).

Another noticeable effect was observed when plants were exposed to a high salt concentration: while all the analyzed clones showed methylation under lack of stress, some of them resulted completely unmethylated under a high salt concentration. For total root, a 25% of the analyzed clones (three out of the twelve) were completely unmethylated, while this amount rose to 75% (nine out of twelve) in the case of epidermis. An unmethylated subpopulation of clones was also detected under mild saline stress but only in the epidermis (displaying seven of the twelve not showing methylation at all).

**Figure 3:**
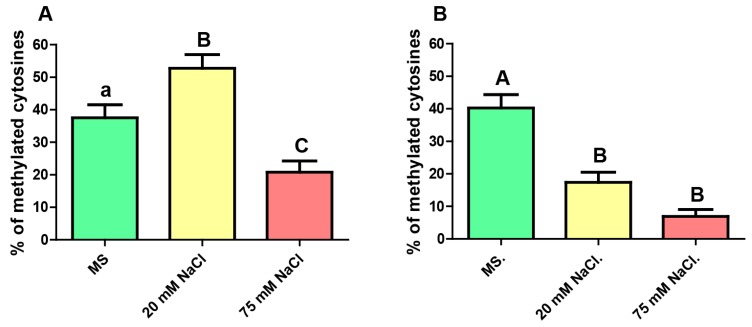
Level of CG methylation (as a% of methylated cytosines) of the analyzed portion of the gene GL2 in roots of plants grown with no stress (MS), mild-saline stress (20 mM NaCl) and severe-saline stress (75 mM NaCl), for (**A**) total root and (**B**) epidermis. Kruskal–Wallis test and Dunn’s post-tests. The columns labeled with the same letter show non-significant differences. Different letters indicates statistically significant differences. At least one small letter indicates *p* < 0.05; one small letter in italics and bold indicates *p* < 0.01. A couple of capital letters indicates *p* < 0.001. The error bars indicate standard error of the mean (SEM).

With the results obtained, we calculated the level of cytosine methylation under the three growth conditions, considering only the CG context (Figure 3).

For mild saline stress, the epidermis revealed a significantly decrease in the CG methylation level, 17 % vs 40 % (*p* < 0.001) in the non-stressful conditions, while whole root showed an upwards trend from 38% to 53% (*p* < 0.05). Analyzing the results obtained for the severely stressed plants it results clear that, no matter which root tissue was analyzed, the level of CG methylation decreased significantly as a consequence of the high salt concentration. In the case of the epidermis, methylation level fell down from 40% to 7% (*p* < 0.001) and for the whole root, it dropped from 38% to 21% (*p* < 0.05). When comparing the two degrees of stress, the analysis revealed a significant difference between plants exposed to 20 mM vs. 75 mM NaCl, but only in whole root.

### 2.2. Phenotypic Studies Related to the Number of Trichoblasts under Stress Conditions 

**Figure 4:**
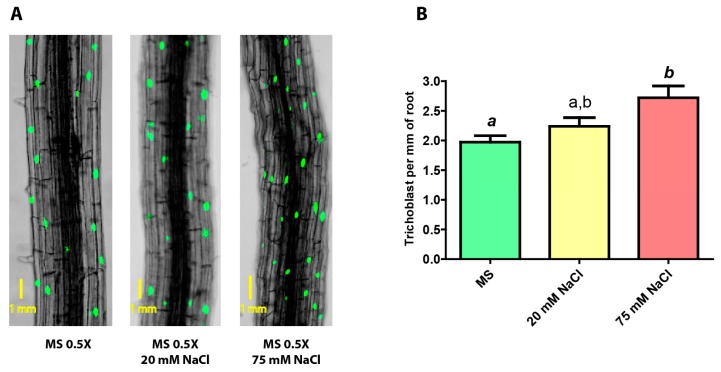
(**A**) Confocal images of roots of pExp7::GFP transgenic plants subjected to growth in no-stress, mild saline stress (20 mM NaCl), and severe saline stress (75 mM NaCl) conditions (A = 100X). (**B**) Number of trichoblasts present per mm of the principal root in pExp7::GFP plants grown under no stress, mild saline stress and severe saline stress conditions. One-way ANOVA and Bonferroni post-test. The columns labeled with the same letter show non-significant differences. Different letters indicates statistically significant differences. At least one small letter indicates *p* < 0.05; one small letter in italics and bold indicates *p* < 0.01. A couple of capital letters indicates *p* < 0.001. The error bars indicate standard error of the mean (SEM).

Next, we analyzed whether the roots of stressed plants showed an altered epidermal phenotype in terms of the number of trichoblasts they developed. We also wanted to investigate if a correspondence could be found between the phenotype and the methylation under the three different growth conditions. With this purpose, growth assays were performed in the same manner as for the other analyses, but in this case, seeds from the pExp7::GFP line were used. This transgenic line, carrying the GFP sequence downstream of the trichoblast-specific promoter of the Expansin7 gene, allowed for visual discrimination and counting of this type of cells. With the aid of a confocal microscope, the number of trichoblasts per millimeter of the primary root was calculated (Figure 4).

It can be seen that the density of trichoblasts in the root slightly increases as the salt concentration rises, from 2.0 in non-stressed plants to 2.4 and 2.8 in mildly and severely stressed plants, respectively; the latter being significantly different compared to non-stressed plants.

### 2.3. Transgenerational Inheritance of the Epigenetic Marks Acquired by Stress

**Figure 5:**
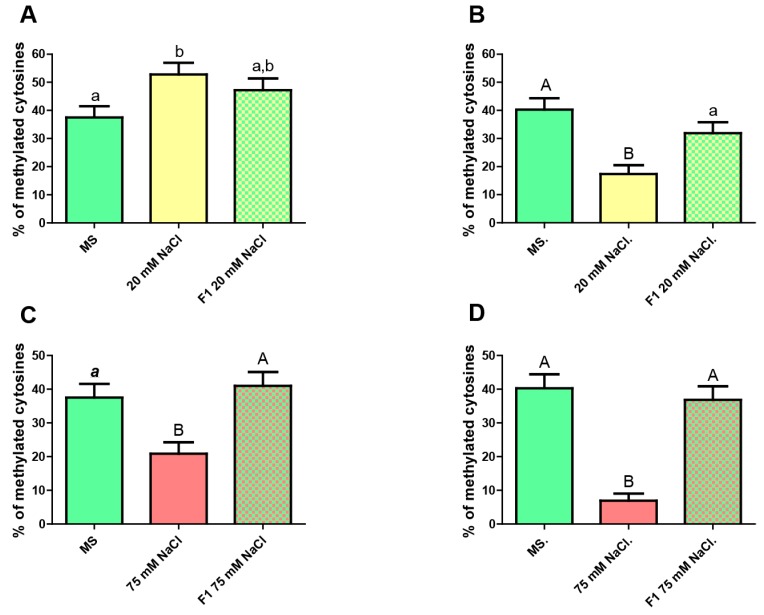
CG methylation level in roots from the unstressed progeny of stressed plants. (**A**) Whole root. From left to right: unstressed F0 (MS), mildly stressed F0 (20 mM NaCl), unstressed F1 progeny of mildly stressed F0. (**B**) Epidermis only. From left to right: unstressed F0 (MS), mildly stressed F0 (20 mM NaCl), unstressed F1 progeny of mildly stressed F0. (**C**) Whole root. From left to right: unstressed F0 (MS), severely stressed F0 (75 mM NaCl), unstressed F1 progeny of severely stressed F0. (**D**) Epidermis only. From left to right: unstressed F0 (MS), severely stressed F0 (75 mM NaCl), unstressed F1 progeny of severely stressed F0. Kruskal–Wallis Test and Dunn’s post-tests. The columns labeled with the same letter show non-significant differences. Different letters indicates statistically significant differences. At least one small letter indicates *p* < 0.05; one small letter in italics and bold indicates *p* < 0.01. A couple of capital letters indicates *p* < 0.001. The error bars indicate standard error of the mean (SEM).

As the inheritance of epigenetic marks is a well proved phenomenon in plants [19], it seemed interesting to us to investigate whether the stress-driven changes in the methylation marks on GL2 could be transmitted to the progeny in a Lamarckian fashion. We performed the assays by germinating and growing the parental individuals in a medium supplemented with 20 mM or 75 mM NaCl and later letting the F1 generation (from self-pollinated stressed F0 plants) germinate and grow under no stress. The results of the methylation analysis for roots from both generations are shown in Figure 5. Kismeth dot plots [20] are shown in Figures S1 and S2.

The results show that total unstressed F1 roots possess a level of CG methylation intermediate between that of the parental plants grown in MS medium alone and those in the same medium supplemented with 20 mM NaCl, with no significant differences respect to any of both. These data do not allow us to conclude if the change in the methylation pattern owing to stress is inherited or not. However, when a similar assay was performed for epidermis, the F1 revealed significant differences comparing to mildly stressed plants but not when comparing to unstressed ones, implying that no inheritance of the methylation pattern has occurred.

When the effect of high salt concentration was analyzed, we observed that F1 plants portray methylation levels similar to those in the unstressed parental individuals and very different from levels found in the stressed parents. This finding was the essentially same regardless of discriminating or not epidermis from total root.

### 2.4. Abundance of Trichoblasts in the Progeny of Salt-Stressed Plants

**Figure 6:**
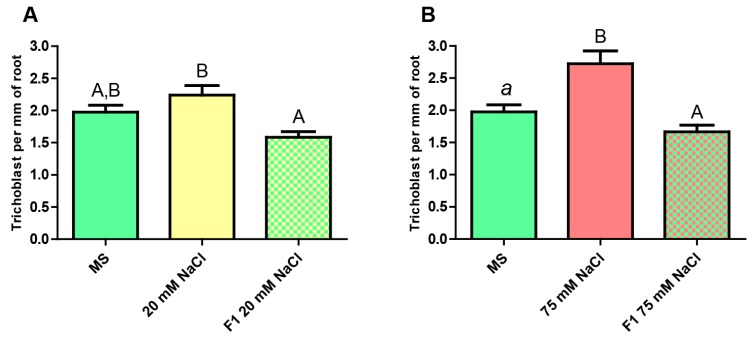
Estimation of the number of trichoblasts present per mm of principal root in pExp7::GFP plants. (**A**) From left to right: unstressed F0 (MS), mildly stressed F0 (20 mM NaCl), unstressed F1 progeny of mildly stressed F0. (**B**) From left to right: unstressed F0 (MS), severely stressed F0 (75 mM NaCl), unstressed F1 progeny of severely stressed F0. One-Way ANOVA and Bonferroni post-test. The columns labeled with the same letter show non-significant differences. Different letters indicates statistically significant differences. At least one small letter indicates *p* < 0.05; one small letter in italics and bold indicates *p* < 0.01. A couple of capital letters indicates *p* < 0.001. The error bars indicate standard error of the mean (SEM).

Finally, we evaluated the phenotype of the F1 individuals derived from plants that had been grown under conditions of either mild or severe stress by estimating the amount of trichoblasts present in the main root (Figure 6, Figures S3 and S4).

In both cases, the number of trichoblast in the unstressed F1 plants presents a noticeable difference with respect to that seen in the stressed plants, whereas no significant differences are observed compared to the unstressed ones.

## 3. Discussion

In this work, we demonstrate that saline stress causes changes in the level of CG methylation of the GL2 gene in its body region. In the epidermis, both mild and severe sodium chloride concentrations caused a decrease in the methylation level. This finding is likely to be linked to lower gene expression levels as CG methylation on the gene body usually leads to higher transcription rates [9]. In this context, lower GL2 expression would result in an increase in the number of trichoblasts, capable of producing root hairs [1]. We were unable to detect GL2 mRNA but our results on the amount of trichoblasts in stressed plants support this scenario.

In an osmotically unfavorable environment, plants are faced with a compromised situation: on one hand, water uptake becomes more difficult, in addition to the tendency to lose it to the more negative water potential of the surrounding environment (with the risk of losing turgor) [15]. On the other hand, the attempt to increase the water uptake would entail a higher flow of solute inside the cell that could bring about ion toxicity.

Another point to be considered is the effect of salt concentration. In the epidermis, there seems to be no effect of the dose on CG methylation. However, when considering the entire root, methylation levels vary significantly in mild vs. severe stress. While the former causes a slight increase, the latter causes a strong decrease. These findings could indicate that the GL2 gene could have additional functions in the root tissues other than epidermis, beyond differentiation of epidermal cells [5,21], perhaps requiring a distinct expression level.

Analysis of the dot plots [20] showed a subpopulation of completely unmethylated clones in the epidermis under both stress conditions, which is not observed under the non-stressful ones. This result is striking, since each clone represents a single DNA molecule (one epiallele). In this context, it is possible to speculate that the two cellular types that compounds the epidermis (trichoblasts and atrichoblasts) respond differentially to stress stimuli, one of them reaching a complete unmethylated status. We also showed specific cytosines that consistently lost their methylation in the epidermis as a consequence of the severe stress applied. This finding is interesting as changes in the methylation status of particular cytosines are reported to correlate with changes in chromatin plasticity, gene regulation and splicing [22].

Evidence on methylation changes provoked by salt stress in plants has been reported [23,24]. However, it is pertinent to discuss the fact that in this work, plants grew under stress conditions from the germination of the seed until three-week-old seedlings. Alternative experimental designs elsewhere consist of seeds germinating first in a stress-free environment and then transferred to the stressing medium of choice [25,26]. Therefore, discrepancies between research groups might arise. For example, our study covers not only the vegetative growth under saline stress but also the germination process, which can be affected by salinity conditions [27], and the early stages of development of the embryo and the root.

As far as the amount of trichoblasts is concerned, the observed increment can be interpreted as a morphological response of the organism to the stressful saline condition. The existence of more hairs might facilitate water uptake from a medium osmotically unfavorable. 

It has been reported that methylation marks acquired by stress in adult individuals before flowering can be transgenerationally inherited in a Lamarckian way [19,28,29] albeit the effect in wild type plants tends to be lost across subsequent generations [30]. However, when we explored if that is the case for GL2 cytosine methylation, salt stress-driven molecular modifications did not happen to pass on to the non-stressed progeny. We cannot rule out the possibility that CG methylation marks returns to basal levels because of the transferring of the parental plants from a stressful environment to a non-stressful one (pots with soil) to be able to achieve seeds. Nevertheless, in some works, DNA methylation in a CG context appears as a stable mark when plants go through a period without stress until production of seeds [31,32]. Finally, the lack of inheritance of abundance of trichoblasts in the root of the unstressed F1 progeny is consistent with the lack of inheritance of changes in epigenetic marks.

## 4. Materials and Methods

### 4.1. Plant Growth

Seeds of wild type *Arabidopsis thaliana* Col-0 plants were sterilized by soaking it on a 30% bleach solution for 12 min and then washed three times with distilled water. Plants were grown on vertical plates containing half-strength MS medium plus 1% agar. For saline stress treatments, media were supplemented with either 20 or 75 mM sodium chloride. After three weeks, roots were cut off for DNA extraction. For inheritance studies, after three weeks of growth in stressful medium, plants were transplanted to pots with a commercial substrate for plants and then watered twice a week. Seeds were collected and after a week sterilized as indicated above. Plants were grown without stress on half-strength MS plates for three weeks, time at which roots were cut for epigenetic experiments.

### 4.2. Selection of Sodium Chloride Concentration for Stress Assays

To choose the sodium chloride concentrations to be used in mild and severe saline stress assays, plants were grown in 0.5× MS supplemented with different concentrations of NaCl, from 1 mM to 100 mM. We decided to use 20 mM NaCl for the mild saline stress because that concentration was the maximum one at which the root length phenotype did not happen to be modified. For a severe saline stress, we employed 75 mM NaCl as higher concentrations produce extremely short roots and very low germination rates (undesirable traits for subsequent bisulfite treatments, for which a high amount of starting material is needed).

### 4.3. DNA Extraction and Bisulfite Reaction

DNA extraction was performed using CTAB (Cetyltrimethylammonium bromide) buffer (Merck KGaA, Darmstadt, Germany) and following the Weigel and Glazebrook protocol [33]. DNA was then cut with EcoRV to obtain fragments between 500 and 2000 bp for proper denaturation during the bisulfite reaction, which was performed according to the Frommer’s lab protocol [34] with the modifications introduced by Gonzalez et al. [12].

### 4.4. Primers Design and Post-Bisulfite PCR

GL2- and Atp1-specific primers were designed (Table 1). For GL2, an epidermal-specific and a general primer were developed. Atp1 was used as a bisulfite-conversion control as this is a mitochondrial gene that lacks methylation [35]. Primers were designed following Wojdacz recommendations [36] and fine-tuned using the Beacons Designer software. Epidermis-specific primers were designed as described in Beyrne et al. [18]. Post-bisulfite PCR was performed in a Techne TC-512 thermocycler (Cole-Parmer, Staffordshire, UK) with the following program: an initial denaturation step at 94 °C for 5 min; 40 cycles of denaturation at 94 °C for 30 s, annealing at 57.3 °C for GL2 epidermis-specific primers, 55.1 °C for GL2 general primers or 62.5 °C for control Atp1 primers; and elongation at 72 °C for 90 s. A final elongation step was performed at 72°C for 5 min. All PCR reactions were run in a final volume of 25 µL with the following ingredients: 0.625 units of Taq DNA Polymerase, 6 µM magnesium chloride, 0.2 µM dNTPs, 0.2 µM forward primer and reverse primer and 75 ng bisulfite-treated DNA.

### 4.5. Sub-Cloning and Sequencing

The obtained PCR products were purified by agarose electrophoresis and appropriate band excision. A Qiagen Gel Extraction Kit was employed (Qiagen, Hilden, Germany). Purified PCR products were cloned into a pGEM-T easy vector (Promega, Madison, Wisconsin, USA) to transform a DH5 alpha *E. coli* strain following the Gonzalez et al. [12] procedure. Positive colonies were selected by growing the sample on ampicillin, IPTG (Isopropil-β-D-1-tiogalactopiranósido) and X-Gal (5-bromo-4-chloro-3-indolyl-β-D-galactopyranoside) plates (Both from Promega, Madison, Wisconsin, USA). Only white colonies were chosen, and the presence and length of the insert of interest was checked by colony PCR using SP6 and T7 promoter universal primers. From positive colonies, a plasmidic DNA extraction assay was performed using PuriPrep-P kit (Inbio Highway, Tandil, Argentina). Plasmids were sequenced using the SP6 universal primer through the services of Macrogen Inc., Seoul, Korea.

### 4.6. Methylation Data Analysis

The obtained sequences were analyzed using the Kismeth software [20] to assess the methylation status of cytosines. For the statistical analysis, the GraphPad Prism (version 5.00) software was employed, using the Kruskal–Wallis test and Dunn’s post-tests for all the comparisons between treatments.

### 4.7. pExp7 Plants

pExp7::GFP plants [37] express the GFP protein but only in the trichoblast cells, readily allowing for visual discrimination of trichoblasts from atrichoblasts in the epidermal layer. These plants were grown under the same conditions described before for WT plants.

### 4.8. Confocal Microscope Imaging

An Olympus FV300 microscope (Olympus Corporation, Tokyo, Japan) was employed. Images were captured using a UPLFL 10X objective with a confocal aperture of 3. The settings used were: EGFP (Enhanced Green Fluorescent Protein) dye with a PMT of 700V, gain 4.0X and an offset of 10%. The transmitted light parameters were set as follows: PMT 215V, gain 3.0X and an offset of 0%. A single image per analyzed root was captured, thus each image represents a different plant.

### 4.9. Image Analysis

In each image, the number of GFP-positive cells was counted and the length of the root portion photographed was also measured, using the ImageJ software. Thus, the number of GFP-positive cells-to-root length ratio was calculated. The value for each treatment was analyzed using the GraphPad Prism (Version 5.00) software. For comparisons between treatments, a one-way ANOVA test was performed with the Bonferroni post-tests.

## Figures and Tables

**Table 1:** Primers employed for post-bisulfite PCR.

Amplicon	Forward Primer	Reverse Primer
GL2 (whole root)	5ʹ-TGAAGATGGTTTAGAGA ATGAT−3ʹ	5ʹ-TAACAAAACATCCCAC TAATA-3ʹ
GL2 (epidermis only)	5ʹ- GGTTCGATATTGGGTC GTTAT-3ʹ	5ʹ-GAACACCAACTATATCGTATTT ATATCTACAAAAACG−3ʹ
Atp1 (for bisulfite- conversion control)	5ʹ-TGAGTAAAGATGTGTTGA AGTGAAAGTT−3ʹ	5ʹ-ACTACCTACACCATACTAATCC AATCA−3ʹ

## Supplementary Materials

The following are available online at https://www.mdpi.com/1422-0067/20/18/4446/s1.
