# Supplementary material for: Effect of Salt Stress on Cytosine Methylation within GL2, An Arabidopsis thaliana Gene Involved in Root Epidermal Cell Differentiation. Absence of Inheritance in the Unstressed Progeny
Source: Int J Mol Sci. 2019 Sep 10;20(18):4446. doi: 10.3390/ijms20184446 (PMC6769687; doi:10.3390/ijms20184446)
Supplement: Supplementary file 1 [file ijms-20-04446-s001.pdf]

Supplementary material

A - Whole root

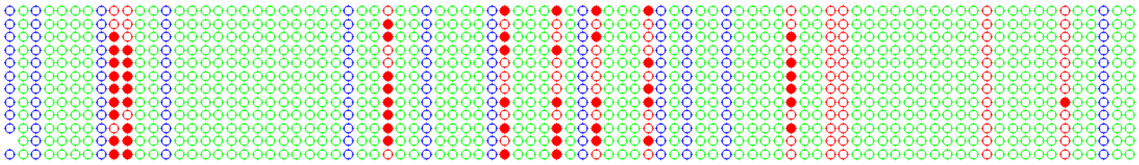

Non-stressed plants

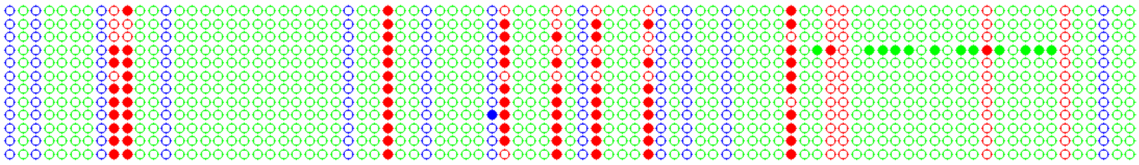

Mild saline-stressed plants

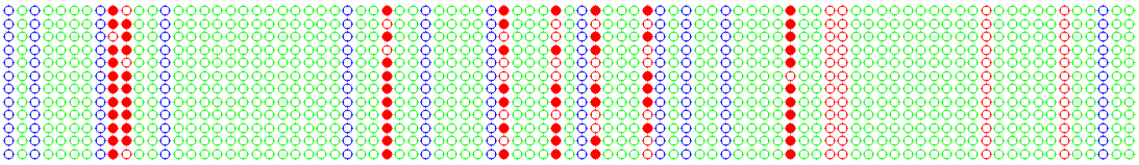

Non-stressed progeny of mild-saline stressed plants

B - Epidermis only

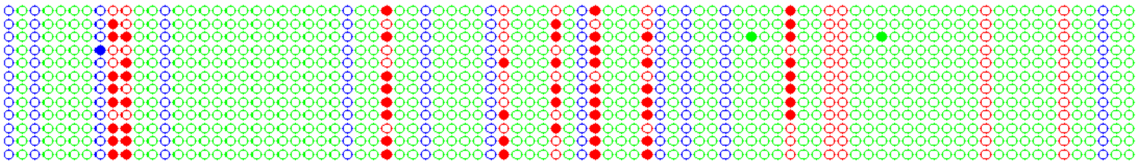

Non-stressed plants

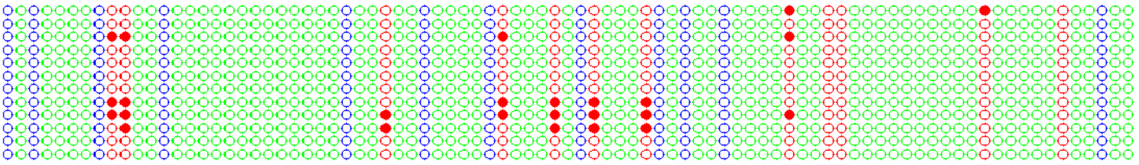

Mild saline-stressed plants

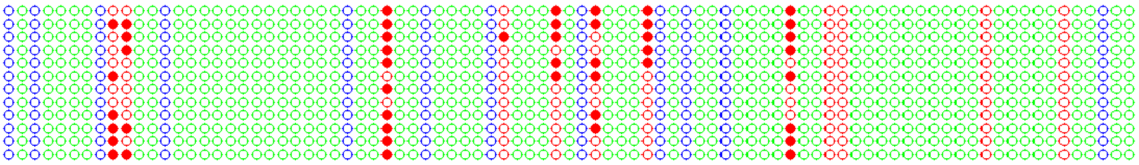

Non-stressed progeny of mild-saline stressed plants

Figure S1. Kismeth dotplots (Gruntman et al. 2008) showing the methylation pattern of a region of Gl2 in the root of non-stressed, mild-stressed and the non-stressed progeny of mild-stressed plants. **A) Total root. B) Epidermis.** Each line represents one clone, and each circle, one cytosine according to the order 5´- 3´ (left - right) in which it appears in the DNA sequence. The colors blue, red and green correspond to cytosines located in the contexts -CHG-, -CG- and – CHH-, respectively. The filled circles indicate methylated cytosines, while the empty circles represent the unmethylated ones.

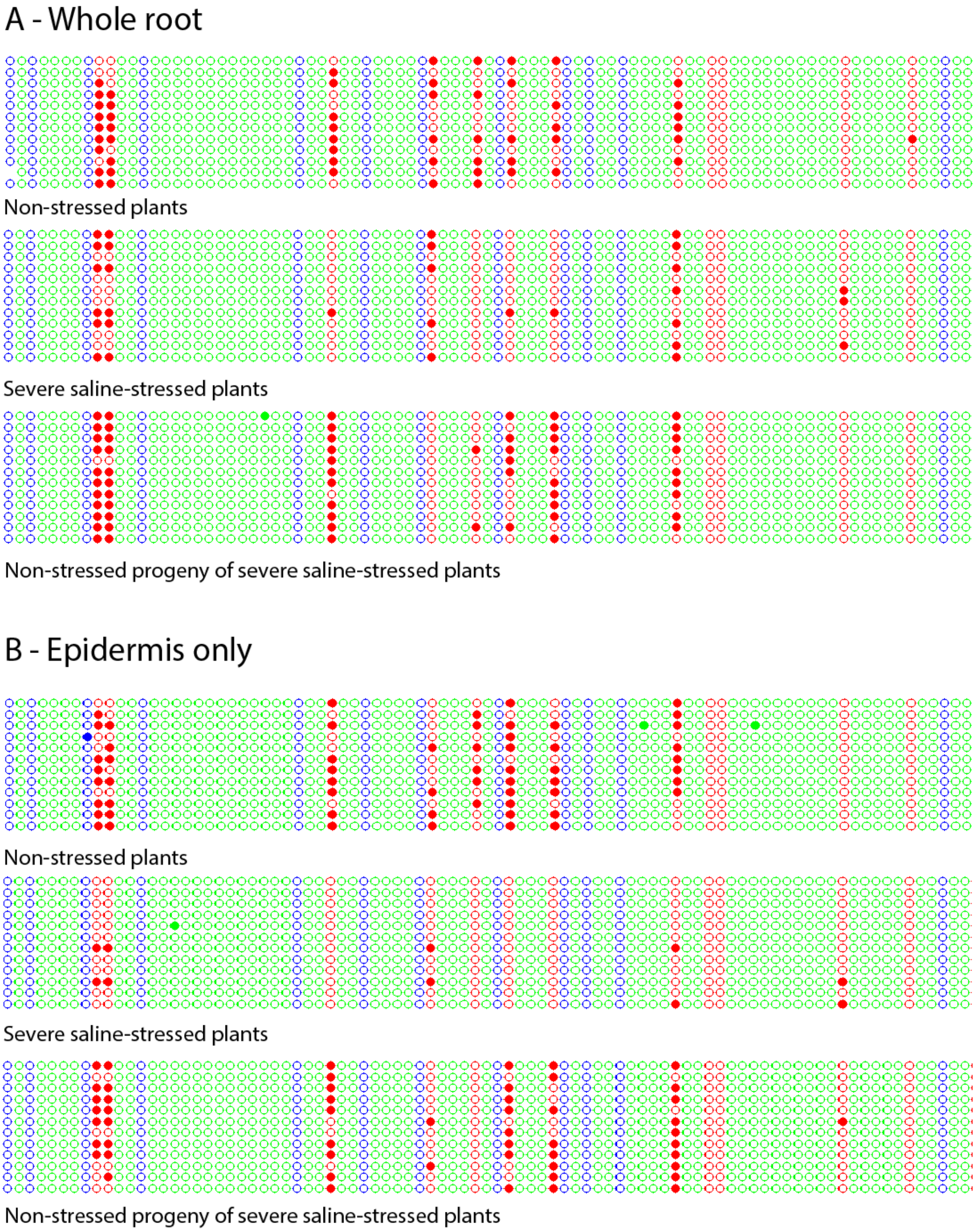

Figure S2. Kismeth dotplots (Gruntman et al. 2008) showing the methylation pattern of a region of Gl2 in the root of non-stressed, severe-stressed and the non-stressed progeny of severe-stressed plants. **A) Total root. B) Epidermis.** Each line represents one clone, and each circle, one cytosine according to the order 5´- 3´ (left - right) in which it appears in the DNA sequence. The colors blue, red and green correspond to cytosines located in the contexts -CHG-, -CG- and – CHH-, respectively. The filled circles indicate methylated cytosines, while the empty circles represent the unmethylated ones.

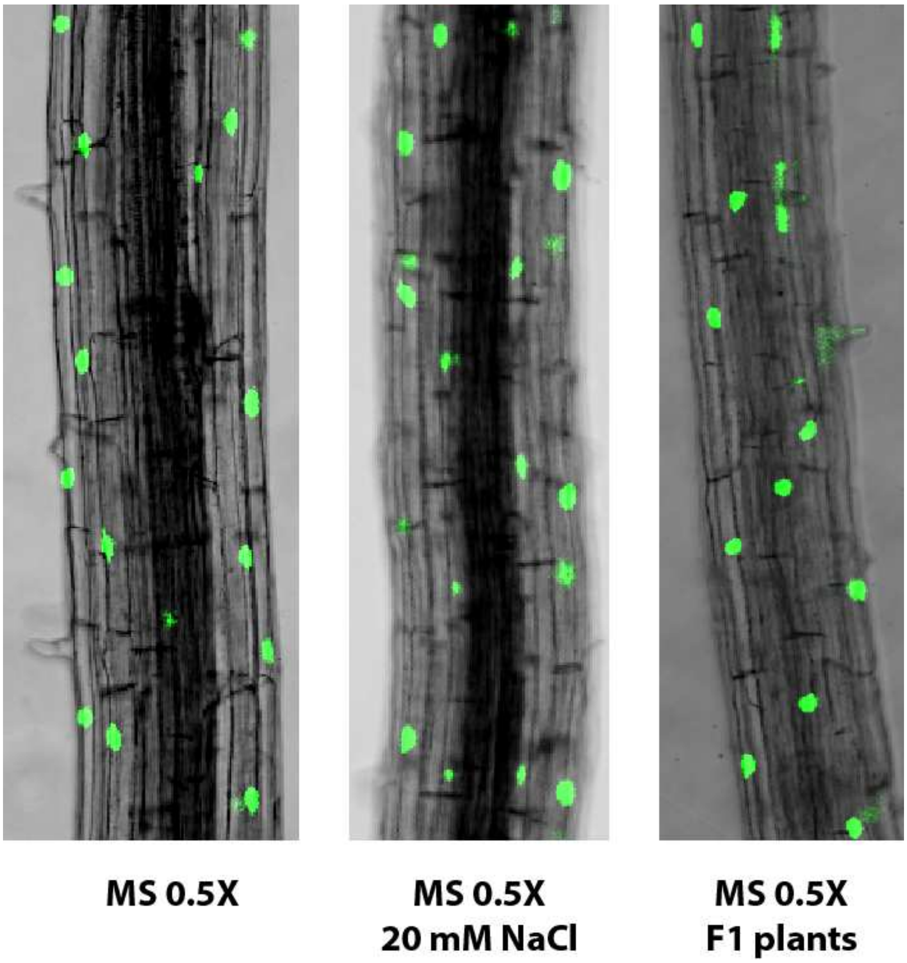

Figure S3. Confocal images of roots of pExp7::GFP transgenic plants. From left to right: non-stressed plants, mild-saline (20 mM NaCl) stressed plants and the non-stressed progeny of mild-saline (20 mM NaCl) stressed plants (A = 100X).

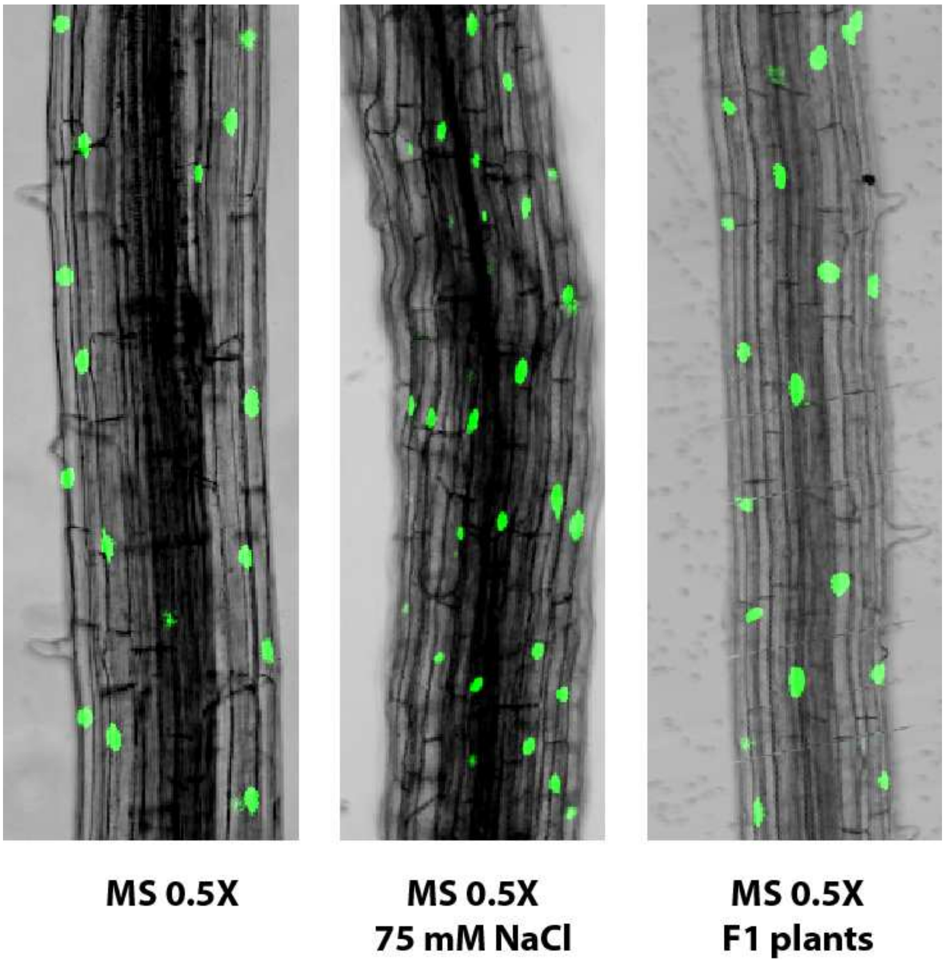

Figure S4. Confocal images of roots of pExp7::GFP transgenic plants. From left to right: non-stressed plants, severe-saline (75 mM NaCl) stressed plants and the non-stressed progeny of severe-saline (75 mM NaCl) stressed plants (A = 100X).
